# Establishing voluntary certification of community health workers in Arizona: a policy case study of building a unified workforce

**DOI:** 10.1186/s12960-020-00487-7

**Published:** 2020-06-26

**Authors:** Maia Ingram, Samantha Sabo, Floribella Redondo, Yanitza Soto, Kim Russell, Heather Carter, Brook Bender, Jill Guernsey de Zapien

**Affiliations:** 1 University of Arizona College of Public Health, 1295 N. Martin Ave, Tucson, Arizona 85724 United States of America; 2 Northern Arizona University, Center for Health Equity Research, 1395 S. Knoles Dr, Flagstaff, AZ 86011 United States of America; 3 Arizona Community Health Worker Association, 424 N. Christine Ave, Douglas, AZ 85607 United States of America; 4 Arizona Department of Health Services, 150 N. 18th Ave, Phoenix, AZ 85007 United States of America; 5 Arizona Advisory Council on Indian Health Care, 141 E Palm Ln, Suite #8, Phoenix, AZ 85004 United States of America; 6 Hualapai Tribe, Hualapai Health-Education and Wellness, 488 Hualapai Way, Peach Springs, AZ 86434 United States of America

**Keywords:** Community health worker, *Promotoras de salud*, Community health representatives, Voluntary certification, Health disparities, Public health workforce, Policy, Scope of practice, Coalition

## Abstract

**Background:**

Community health workers (CHWs) are widely recognized as essential to addressing disparities in health care delivery and outcomes in US vulnerable populations. In the state of Arizona, the sustainability of the workforce is threatened by low wages, poor job security, and limited opportunities for training and advancement within the profession. CHW voluntary certification offers an avenue to increase the recognition, compensation, training, and standardization of the workforce. However, passing voluntary certification legislation in an anti-regulatory state such as Arizona posed a major challenge that required a robust advocacy effort.

**Case presentation:**

In this article, we describe the process of unifying the two major CHW workforces in Arizona, *promotoras de salud* in US-Mexico border communities and community health representatives (CHRs) serving American Indian communities. Differences in the origins, financing, and even language of the population-served contributed to historically divergent interests between CHRs and *promotoras*. In order to move forward as a collective workforce, it was imperative to integrate the perspectives of CHRs, who have a regular funding stream and work closely through the Indian Health Services, with those of *promotoras,* who are more likely to be grant-funded in community-based efforts. As a unified workforce, CHWs were better positioned to gain advocacy support from key health care providers and health insurance companies with policy influence. We seek to elucidate the lessons learned in our process that may be relevant to CHWs representing diverse communities across the US and internationally.

**Conclusions:**

Legislated voluntary certification provides a pathway for further professionalization of the CHW workforce by establishing a standard definition and set of core competencies. Voluntary certification also provides guidance to organizations in developing appropriate training and job activities, as well as ongoing professional development opportunities. In developing certification with CHWs representing different populations, and in particular Tribal Nations, it is essential to assure that the CHW definition is in alignment with all groups and that the scope of practice reflects CHW roles in both clinic and community-based settings. The Arizona experience underscores the benefits of a flexible approach that leverages existing strengths in organizations and the population served.

## Background

Public health leaders have issued extensive calls to action on health equity and the social determinants of health [1–3]. There is a strong evidence base for the effectiveness of community health workers (CHW) workforce in achieving health outcomes [4–6], and the workforce is frequently incorporated into public health strategies to address health disparities. CHWs are known for their enduring front-line efforts to connect vulnerable populations to support services and represent their communities in overcoming systemic barriers to optimizing health [7–9]. There is little attention, however, on the financial stability and professional mobility of CHWs themselves. Fair and decent employment is a key recommendation in the World Health Organization’s Commission on the Social Determinants of Health [3]. In the US, recognition of, and compensation for, the professional status of CHWs is essential to bolster and reinforce their vital role in the public health and health care delivery systems. In this article, we describe how the Arizona CHW workforce, made up largely of *promotoras de salud* working in the U.S.-Mexico border region and community health representatives (CHRs) serving American Indian Tribes and communities, worked in parallel and collaboratively to establish CHW Voluntary Certification as a means to elevate their status and establish their credibility as a professional workforce.

Across the US and globally, there is growing interest in specific policies designed to increase the role of CHWs in the health care system in order to improve health care access and decrease health inequities, as well as lower costs [8, 10]. Reviews of evidence-informed CHW policies designed to improve population health designated CHW core competency certification as among the best approaches both in the US and globally [11], with documented improvements in diabetes-related outcomes and increased access to services [12, 13]. Importantly, CHW certification will contribute to a common understanding of the role and unique contribution of CHWs in the health care system, which can facilitate reimbursement mechanisms with increased compensation and job security [10]. However, some states have experienced difficulties in determining how to define and develop the CHW workforce, particularly in the absence of a state-supported CHW program [14]. Involvement of CHWs in the development of the certification policy is a key recommendation across states that are working on CHWs policies, and this can be achieved through the establishment of CHW professional organizations and the support of state-level CHW programs. While documentation of policy initiatives is a moving target, prior to the passage of the Arizona CHW voluntary certification described in this paper, six states in the United States of America had passed CHW certification laws, Massachusetts, New Mexico, Maryland, Ohio, Oregon, and Texas, and five of those states required involvement of CHWs in developing certification requirements [12, 15]. With the exception of Texas, all states have voluntary certification. Notably, policy reviews focus on the evidence base for health outcomes and do not address opportunities for increased compensation and mobility of individual CHWs.

## Case description

On May 16, 2018, Arizona’s 23rd Governor Douglas Ducey signed the CHW Voluntary Certification HB2324 legislation into law. The bill’s passage represented another step in a long journey initiated by the Arizona CHW Association (AzCHOW) to organize, support, and build recognition for CHWs. It also represented the unification of the two major CHW workforces in the state, *promotoras* and CHRs. Differences in the origins, financing, and even language of the population-served contributed to historically divergent interests between CHRs and *promotoras*. The policy development process, which began long before voluntary certification became the desired outcome, clarified to the stakeholders that both groups shared the same profession, encountered many of the same challenges, and most importantly, had greater political power when they worked together. In fact, in a state reluctant to approve any new regulation [16], it is unlikely the legislation would have passed without unified support of the *promotora* workforce and Tribal CHR programs. In this paper, we seek to elucidate the lessons learned in our process that may be relevant to CHWs representing diverse communities across the a US and beyond. We focus on aspects of the policy development and advocacy process to underscore key decisions that contributed to successful passage of the law.

### The community health worker workforce in Arizona

A sister state to Sonora, Mexico and a geographic region populated by 21 federally recognized American Indian Tribes with Reservation lands, Arizona has a rich and diverse cultural heritage. Tribal reservations make up over a quarter of Arizona’s land base, and Arizona has the 3rd largest American Indian population of any state, comprising 5–6% of Arizona’s total population [17]. One quarter of Arizona’s residents identify as Latino, the majority of whom are concentrated in US-Mexico border counties and are of Mexican origin [18]. While this diversity is an asset, the health of the state is challenged by political, economic, and social conditions that disproportionally affect American Indians and Latinos [17, 19].

As in many states, CHWs have a historical and ongoing role in addressing Arizona’s health disparities and specifically in building bridges between health and human services and vulnerable communities [20]. Unfortunately, and perhaps reflective of bias within the health system, CHWs earn only minimum wage on average in Arizona and many are grant funded and have little job security [21]. Very few organizations that hire CHWs have internal structures that ensure ongoing training or support CHW promotion within the job CHW designation. Further, no external accrediting structure exists through which a CHW’s work experience is recognized financially as she moves from one organization to another. It is thus imperative to improve working conditions for CHWs in Arizona as they are increasingly called upon to solve health care’s most pressing health issues both in the state and across the nation [8]. One strategy for improving the compensation, mobility and sustainability of the workforce is to formally assess and recognize core competencies and scope of practice through a state-administered certification program.

CHRs were among the first CHWs in Arizona. In the 1960s, American Indian communities in the US identified the need and advocated for community health professionals that would improve cross-cultural communication between American Indian communities and predominantly non-American Indian health care providers. This advocacy led to the emergence of a federally funded CHR program. In 1969, Congress appropriated funds for the CHR Program as a component of health care services of American Indian people [22]. The CHR Program is appropriated funding by Congress every year and is administered by the federal Indian Health Service (IHS). Most CHR programs are contracted/compacted by Tribes from the IHS. The CHR programs direct well-trained, community-based, health care professionals, designed to integrate the unique support of Tribal life with the practices of health promotion and disease prevention. The CHR workforce acts as a liaison and advocate for clients to assist them in meeting their health care needs, while upholding traditions, values, language and cultural beliefs of the individuals they serve [23]. Existing literature on CHR programs focuses on evolving roles of CHRs [24] and the evaluation of CHR training programs on health conditions [25–27]. Of the 22 federally recognized Tribes in Arizona, 19 Tribes operate a CHR Program.

*Promotora* programs in Arizona originated from an academic-community partnership in the 1980s that developed a CHW prenatal intervention in a US-Mexico border community called *Comienzo Sano (*Health Start) [20, 28, 29]. The intervention was eventually adopted as an evidence-based program by the Arizona Department of Health Services (ADHS) and is now delivered by CHWs in county health departments and agencies across the state to provide services to rural and underserved mothers and children. Over the next decades, the partnership continued to champion the CHW/*promotora* workforce in the border region, documenting effectiveness in cancer prevention [30], chronic disease prevention and management [31–33], and public health policy change [20, 34]. Federally qualified health centers (FQHCs) and community-based organizations were pivotal in identifying grant opportunities and designing *promotora*-driven programs [32, 33, 35, 36]. The need to secure grant funding, while destabilizing for the programs, and the CHW/*promotoras* working in them, also contributed to developing a strong evidence-base for CHW programs because they required rigorous evaluation. Notably, the *promotora* workforce originated in a community-based model [28] in which they employed an array of methods, including community organizing, to champion the needs of marginalized populations such as farmworkers [20]. Growing evidence led to proliferation of CHW/*promotora* programs beyond the border region, as well as their integration into primary care service delivery [37], both as part of care teams and through community–clinical linkage models [38]. However, the prevailing issue of workforce sustainability remained largely unaddressed.

The emergence of the Arizona Community Health Worker Association (AzCHOW) was essential to the development of the CHW workforce. As an organization of, by, and for CHWs, AzCHOW was formally established in 2001 by a group of CHWs who identified the need to create a forum to inform and unite culturally diverse CHWs of all disciplines and to strengthen the professional development of the CHW workforce through training, resource sharing, and collaborative opportunities with community, government, health, and educational institutions. Over 20 years, AzCHOW engaged in activities designed to cultivate a collective voice for CHWs in addressing relevant policy and sustainability issues, provide appropriate training opportunities, and promote expansion of the CHW workforce. CHRs served on the board of AzCHOW and participated in training events; however, full representation of this workforce in statewide initiatives was historically lacking. As a CHW-driven organization, however, AzCHOW was the natural leader to bring CHRs and *promotoras* together to consider statewide CHW voluntary certification.

## The pathway to CHW unification and voluntary certification

In 2013, the Arizona Department of Health Services (ADHS) contracted with an academic institution to convene approximately 15 CHWs and their stakeholders to explore strategies to sustain the workforce, as well as identify approaches to integrate CHWs across the health and public health delivery systems. The group evolved over time to include additional stakeholders and formalize itself as the Arizona Community Health Worker Workforce Coalition (see Table 1, timeline). In considering certification, a major point of concern was that a central characteristic of an effective CHW, identification and allegiance to the community, might be compromised by professionalization because it would define expertise by competencies rather than by inherent personal qualities. Our discussion reflected the national dialogue regarding certification [40] and underscored the importance of CHW leadership developing any future legislation. From its initiation, AzCHOW played a major role in the coalition in representing the interests of CHWs and elevating their voice in decision-making processes, either by bringing them directly to the table or by gathering their perspectives through small group sessions and annual meetings.

**Table 1:** CHW voluntary certification timeline

Year	Major events
2013	• CHW Workforce Coalition (convened in 2012) approves CHW definition, core competencies, and scope of practice. • Coalition recommends 10 action steps to ADHS including establishing CHW Program.
2014	• ADHS establishes CHW Program Manager position. • Coalition establishes workforce development and sustainability committees. • Coalition members developed advocacy fact sheets on CHWs.
2015	• Partners meet with Democratic Senator to discuss CHW sustainability; he subsequently hosts a forum to with Medicaid health care plans and insurance companies. • AzCHOW builds consensus among CHWs on certification through state wide survey, focus groups and annual meeting. • Coalition conducts a provider survey on benefits of CHWs. • Partners reach out to AACIHC and ADHS Tribal liaison to discuss CHR workforce. • Coalition develops a Sunrise application for a change in a health care profession scope of practice but decides not to submit it. • Partners hold listening sessions with CHR programs, Tribal Health Department Directors, and American Indian health policy experts. • CHR organize CHR Movement and host first annual CHR Policy Summi with 10 CHR Programs to discuss CHW certification efforts occuring in Arizona and New Mexico.
2016	• Democratic Senator hosts 2nd forum with stakeholders who recommend moving forward with Sunrise application and legislation. • Arizona Alliance of Community Health Centers and Arizona Public Health Association sign policy declarations in favor of certification giving boost to effort. • AzCHOW submits Sunrise application to Health Committee of Reference where it passes, but with opposition. • Hualapai Tribe adopts a tribal resolution to support the CHR workforce. • AzCHOW and CHR Movement discuss certification at annual events. • Coalition develops CHW Core Competency Training Approval Process. • CHR movement hosts second annual CHR Policy Summit with 18 CHR Programs for continual vetting of certification with members.
2017	• AzCHOW holds emergency meeting and decides to pursue legislation. • Democratic Representative sponsors CHW Voluntary Certification Bill. • CHW Bill passes out of the House of Representatives. • Senate Speaker assigns bill to Committee on Trade and Commerce where Chair declines to hear the bill. • AzCHOW receives foundation funding to support voluntary certification efforts. • Coalition members meet with opposition in the Senate to discuss bill. • AzCHOW approves the first CHW core competency training. • CHR Movement hosts third annual CHR Policy Summit with 100+ attendees and Tribal CHR Programs from 7 states.
2018	• Partners meet with Republican Representative and Chair of the Health Committee, who agrees to sponsor the bill. • CHR partners point out the need for reciprocity to be stated in the legislation • Coalition members galvanize broad support for the bill. • CHW/CHR workforce and stakeholder testify in Senate and House committees. • Bill passes the Senate and the House. • May 16, Governor Ducey signs the bill into law. • CHR Movement formalizes a CHR Coaltion meeting monthly on workforce policy issue and annual CHR Policy Summit planning.
2019	• ADHS forms the CHW Advisory Council for guidance on rules. • Advisory Council begins crafting recommendations for CHW definition, core competencies, and training and renewal requirements. • AACIHC commissions academic Coaltion partners to conduct a broad based CHR workforce assessment.

### The Arizona Community Health Worker Workforce Coalition

After a year of discussion and information gathering, the coalition made ten overarching recommendations to ADHS, the most salient of which was to establish a CHW Project Manager within the state health department. Between 2013 and 2014, ADHS procured funds to hire the first CHW Program Manager dedicated to building awareness and partnerships for the workforce at the statewide level. The CHW Program manager worked closely with the AzCHOW and the Coalition, which grew to over 200 individuals and organizations as part of the CHW voluntary certification effort. In a pivotal moment in 2014, the founders of the coalition reached out to the Arizona Advisory Council on Indian Health Care (AACIHC) to gauge interest in CHW/CHR issues. The AACIHC serves as a resource for Tribal governments and the State of Arizona in meeting the unique health care needs of the Arizona American Indian population. This organization had influence and insight that was distinct from the broader workforce as it had a legislative charge to advocate for health policies that would benefit Tribes and tribal health systems. Their incorporation into the effort marked the beginning of CHW unification.

With an overall objective to promote recognition and standardization of the workforce, the CHW Workforce Coalition viewed legislation as the last and least desirable option, particularly in a state where legislators were unlikely to view new regulation of health professions favorably. Stakeholders wanted to be sure that a state law was necessary for reimbursement of CHW services by the Arizona Health Care Cost Containment System (AHCCCS), the body overseeing administration of Medicaid (the US health insurance safety net) in the state. The first 2 years were spent gathering data and gaining consensus on strategies for moving forward. Key efforts included CHW surveys and focus groups to gather perspectives on certification, including with Tribal CHR programs, through a small Area Health Education Grant. The academic partner conducted a statewide provider survey that documented perceptions of the benefits of CHWs on the quality of health care. They also completed a workforce survey designed to estimate the number of CHWs in Arizona, documenting that roughly 30% of the workforce consisted of CHRs.

### The Community Health Representative Movement and Policy Summit

Growing awareness that CHRs made up a substantial sector of the CHW workforce was key in increasing the interest of tribal advocates, not only the AACIHC, but also the Inter Tribal Council of Arizona and CHR Programs representative of Navajo Nation, White Mountain Apache Tribe, Hualapai Tribe among 19 others operating in Arizona. Questions arose about why CHRs were not more integrated in statewide efforts historically, and this critical viewpoint was accompanied by an awareness among CHRs that they needed to engage in organizing efforts. CHRs realized that there was a lack of knowledge among tribal leadership regarding what CHRs do, despite being the oldest CHW program in the nation and serving tribal communities for almost 50 years. This point was particularly salient because Tribes in Arizona have a government-to-government relationship with the state of Arizona, including AHCCCS, ADHS, the state legislature, and the governor. From this point forward, the CHRs began considering their distinct interests in the process.

In 2015, CHR stakeholders organized the first CHR Policy Summit to dialogue and plan for the unique issues and opportunities facing CHR workforce sustainability, recognition, and advancement. Over time, the Annual Policy Summit grew in membership and scope resulting in the CHR Workforce Movement. Members include CHRs and CHR supervisors, health department directors, American Indian health social policy members, and university partners, who continue to advocate for inclusion of CHRs in state- and national-level dialogue and policy regarding workforce standardization, certification, training, supervision, and financing. Like many professional associations and professional conferences, the CHR Summit and CHR Movement provide an interactive environment and mode of continuous communication among stakeholders in which CHW voluntary certification as well as other policy initiatives and advocacy strategies unique to the CHR workforce can be discussed and deliberated.

### Key decisions in the policy development process

In a parallel and overlapping process, key advocates from the CHW Workforce Coalition initiated the path to certification by conferring with a minority party Senator from Southern Arizona with years of experience in building support for public health legislation from the ground up. While not well positioned to sponsor a bill, the Senator was instrumental in hosting stakeholder meetings with key players in the health care policy arena who otherwise would not have found the time-legislative liaisons with AHCCCS and ADHS, health insurance plan representatives, providers, and policy advisors. These meetings, which included *promotora* and CHR representatives, informed a series of key decisions, each of which led to the next step towards voluntary certification. The first was the recommendation that Arizona CHWs continue to develop a voluntary certification process independent from legislative action to demonstrate interest and commitment to certification. The approach also recognized that passage of regulatory legislation was extremely unfavorable. Under AzCHOW’s initiative, coalition members formally adopted the American Public Health Association (APHA) definition of CHWs and finalized the Arizona CHW core competencies and scope of practice based on national standards [39]. AzCHOW and the coalition developed a voluntary certification application process that included a formalized assessment of CHW mastery of core competencies. The assessment was designed as an interactive session between CHWs, a key component of which was to demonstrate their ability to communicate their role as CHWs to other health professionals.

AzCHOW also worked with the coalition to develop a process to approve CHW training programs that adhered to the core competencies and scope of practice adopted by the coalition. The approval process convened a committee made up of three coalition members with at least one CHW and one person from ADHS who would review of curriculum materials and conduct a site visit. In a pilot effort, a community college submitted their curriculum and a committee of coalition members led by AzCHOW evaluated and approved the training program for voluntary certification. This approach was extremely valuable in recognizing that diverse organizations across the state hired and internally trained CHWs, but that they did not necessarily understand, train, or utilize the workforce appropriately. FQHCs, county health departments, community agencies, and community colleges were encouraged to organize and complement their existing training activities to address core competencies comprehensively. A major benefit of this approach was that many CHWs would receive core competency training as part of their job activities and would thus be financially neutral. These activities functionally demonstrated to policy makers that there was an established mechanism in place to prepare and evaluate CHWs, coupled with buy-in from a diverse set of stakeholders on the coalition. Coalition members also recommended that the legislation include “continuing education” requirements of certification to ensure that CHW organizations recognized the need to provide ongoing professional development opportunities to their CHW employees.

The second major decision followed a recommendation of the policy advisors to submit a “Sunrise” application, as per state statute, to the interim health committee of reference (COR), responsible for reviewing proposed changes to the scope of practice for health professions. This process was a unique feature of the Arizona legislative process. The Coalition developed an application but delayed submitting for a year to explore alternatives and build broader consensus. The COR voted to approve the application the following year; however, it was not without opposition from committee members and other health professions. Several COR members expressed concern about establishing a new regulatory requirement for a profession that had previously existed with no license or government oversight. Others were ideologically opposed to new regulation of any kind, and they influenced other COR members to change their vote from “aye” to “no.” The response surprised Coalition members, and they subsequently made efforts to personally meet with every legislator on the health committee, as well as to educate all relevant interest groups, including physicians, nurses, health departments, and FQHCs.

The third major step in the process resulted from clarification by the AHCCCS legislative liaison that state certification would be required in order for Medicaid to reimburse CHWs for their services. Reimbursement was a key component of the CHW sustainability approach. While this reality pushed the coalition towards a legislative solution, AzCHOW and leaders of the CHR Movement continued to weigh the pros and cons of continuing to move forward during that particular legislative session. A crucial component of the legislation was the decision to include a clause that required ADHS to set up a CHW Advisory Council made up of a majority CHWs that would be responsible for recommending the details of the voluntary certification, including the cost to CHWs, finalizing core competencies, and education and renewal requirements. The inclusion of the CHW Advisory Council was key because it meant that the final details of CHW voluntary certification would be controlled by ADHS in the rule making process and based on recommendations from CHWs and their stakeholders, rather than by lawmakers who are less familiar with the workforce and may have political agendas separate from the workforce. After the CHW Advisory Council finalizes recommendations, the rules would become open for public comment prior to enactment. Additionally, the Tribal CHR programs advocated for legislative language detailing reciprocity for voluntary certification, ensuring that CHRs who were certified by Tribes or the IHS would also be certified by the state.

### The role of external stakeholders in the passage of voluntary certification

The CHW Voluntary Certification bill was introduced that year after being approved by the COR. It was introduced in the House chamber and passed by House Health Committee and on the House floor, then transferred to the Senate chamber. However, the bill was assigned to a disinterested committee in the Senate, and the committee chair declined to give it a hearing, despite efforts of the coalition members to advocate with this member. Over the following year, CHWs gained active support from people and groups who were initially “fence sitters”, or organizations who neither opposed nor supported the advocacy effort. The most significant was the decision by a representative from the majority party and Chair of the Health Committee to sponsor the bill in the next session. As Chair, she was able to include the bill in her legislative agenda, which proved to be a critical step in the success of the passage of the bill. Since the corresponding Chair in the Senate had failed to move the bill forward the previous session, now the House Health Chair could help to ensure the bill passed the COR and receive a hearing in the opposite chamber. Efforts by Coalition members to educate key stakeholders on the value of CHWs to the quality, efficiency, and effectiveness of the health care system also garnered the support of influential lobbyists for both the largest health care system in the state and the state association of Medicaid health care plans. 

The major roadblock facing the bill as it moved through the various committees was the question of whether or not voluntary certification was needed, and whether this “regulation” would be restrictive or burdensome to the individuals doing the work. Further, some lawmakers wanted to know that the bill had to be “vetted” by groups apprehensive of governmental regulation, yet completely unrelated to the healthcare industry. The ongoing advice and advocacy from seasoned leaders in the health care industry allowed coalition members to maneuver through and ultimately to overcome the challenges. While the final draft of the bill was less protective of the integrity of the CHW workforce in terms of definitions and training, the inclusion of the CHW Advisory Committee ensured an opportunity to fine tune details of the law during the rule making process that followed its passage into law. Importantly, the final legislation left many details of CHW voluntary certification up to the recommendations of the CHW Advisory Committee and the official rule-making process conducted by ADHS. With the anticipated conclusion of the rules making process in 2020, the Advisory Council recommended the adoption of AzCHOW’s CHW core competency training approval process, a $100 certification fee, and documentation of 12  continuing education hours every 2 years on a wide array of topics.

## Discussion

In setting out to elevate the status of the CHW workforce in Arizona and address sustainability, AzCHOW drew upon successful strategies from other states such as New Mexico and Massachusetts [40], where CHW leadership and broad stakeholder involvement were also key. AzCHOW learned from and mirrored strategies from other states in several essential ways by (1) working hand in hand with the state health department in all steps of the process; (2) building a strong coalition of CHW stakeholders who reached consensus regarding core competencies and scope of practice [41]; (3) working with an academic partner to systematically gather information on the workforce from various perspectives, primarily CHWs, but also health care plans and health care providers; (4) securing financial support from a foundation that allowed AzCHOW to work directly on policy change; and (5) passing legislation that established an advisory council of majority CHWs, responsible for setting up the voluntary certification process [40, 42].

It is notable that Arizona embodies contextual factors that are shared by many states that have moved forward with certification. Arizona is similar to New Mexico in having a large rural population and a geographic region that is populated by Tribal Nations; in fact, the Navajo Nation straddles the two states, underscoring the need for Tribal reciprocity included in the legislation. As a border state, much of Arizona’s Latino population is of Mexican origin, while the Latino population in other states is more diverse and may have distinct concerns. However, Arizona shares a major characteristic with many states of having a large immigrant and undocumented population which can be effectively reached by community health workers [43, 44]. Despite differences, states across the US have expressed interest in CHW certification through a variety of approaches [45].

The case study also provides insight into how to address the challenge of certification in a politically conservative state. Similar to many states, the Arizona legislature has a strong anti-regulatory stance and requirement for budget neutrality (i.e., no fiscal impact to the state budget). The decision-making process to formalize the workforce resulted in strong consensus and solidarity across multiple stakeholders. AzCHOW and the CHW Workforce Coalition members met repeatedly with representatives of large health care delivery systems, FQHCs and Medicaid health plans over the 5 years that culminated in passage of the law, cultivating mutual respect and interest in CHWs as a unique workforce. Ongoing assistance in policy development and direct advocacy efforts from these partners were essential to addressing myriad concerns of the legislative body.

The case study is also relevant to all states that share lands with tribal governments that employ CHRs. By unifying their workforce, dominated by *promotoras de salud*, with a long history of working with Latino and border populations and tribally employed community health representatives, who make up 30% of the state workforce, Arizona CHWs were able to control the legislative process. *Promotoras* specifically brought the advocacy and leadership skills associated with grassroots movements, while CHRs provided a model of training and integration into the formal health care system, as well as a special relationship to and influence on governmental entities.

### Lessons learned from unifying the CHW workforce

The major lesson of this case study is the early engagement and leadership of CHWs in the legislative process. While this has been demonstrated in other states, our experience stresses the *importance of CHR involvement in state discussions of the CHW workforce, an accomplishment* that has not been described elsewhere. There are currently 573 federally recognized tribes across 36 US states, and our case study provides an example not only the importance of unification of CHWs in passing legislation, but also strategies to build a common agenda among CHWs from diverse communities. It is an ongoing challenge to integrate the perspectives of CHRs who have a regular, albeit insufficient, funding stream and work closely within Indian Health Service and Tribal health systems, with those of *promotoras* who are more likely to be sustained by grant funding in community-based efforts. By including Tribes in the legislative advocacy efforts, successful passage of the bill was heightened. The AACIHC was established to give tribal governments, tribal organizations, and urban Indian health care organizations in Arizona representation in shaping Medicaid and health care policies and laws that impact American Indians. Medicaid has played an increasing role in funding health care services to tribal members since passage of the American Indian Health Care Improvement Act [46]. Thus, Tribes may be more interested in and affected by state-level legislative decisions, such as Medicaid expansion. Tribal relationships with state government offer a distinct influence on the state legislative process and may have convinced lawmakers to pay closer attention to the CHW voluntary legislation than they might otherwise have done.

CHW and CHW stakeholders nationwide have expressed interest in the Arizona experience for direction on how to include CHRs in state-level CHW efforts. CHW stakeholders in other states many not know how to work with tribes nor understand that as sovereign nations, Tribes have well-established mechanisms, often by statute to directly advocate and lobby at the local, state, and federal level. Other key partners included regional advocacy and capacity-building organizations, such as the Inter Tribal Council of Arizona, that promote American Indian self-reliance through public policy development. The Arizona case provides a road map for CHW stakeholders to support CHR program managers and CHRs to discuss their needs and priorities as part of the advocacy process. The fact that the CHR Movement is now in its 6th year is evidence of the need for patience and commitment to a process to support this highly valuable, yet undervalued and undercompensated tribal health workforce. We found that CHR Policy summits were effective in validating the work of CHRs and their contribution to the health system.

We recommend that laws enacting CHW voluntary certification provide minimal restrictive guidelines and leave the details of implementation to an Advisory Council with majority CHW involvement. As we move forward in Arizona with the CHW voluntary certification, we are keenly aware of and sensitive to the differences and needs of CHRs and *promotoras*. In making recommendations for implementation of the law, the CHW Advisory Council is essential in assuring that the definition of a CHW is aligned with both groups and that the scope of practice reflects both CHW roles in both clinic and community-based settings. The CHW Advisory Council is also recommending that the guidelines for CHW core competency training developed by AzCHOW be adopted in the rules process, thus capitalizing on this important formative work that was spearheaded by CHWs. To date, three organizations have received CHW Core Curriculum Training approval under the AzCHOW process. A step-by-step guide for organizations interested in preparing their programs for approval is available on-line [47].

Importantly, CHRs and *promotoras* are represented in the Advisory Council. While there are clearly benefits to identifying a collective workforce, distinctions in their respective roles, community served, and services provided may influence decisions about the scope of practice, required training, and professional conduct. Nonetheless, we use our experience to promote the benefits of using CHWs as an umbrella term that can embrace all members of the workforce serving diverse populations. It will be important in moving forward to include representation from CHWs serving other populations. We predict that additional requirements and opportunities for core competency and specialized training across the CHW workforce will contribute to increased capacity and professional growth for all CHWs. Further, the identification of billing codes for CHW services will surely apply to both CHR and CHW services. Perhaps most importantly, a unified workforce will provide a stronger advocacy base when funding sources for CHWs or the populations they serve might be threatened in the future.

## Conclusion

In this article, we share highlights from a long and complicated journey toward legislation to establish CHW voluntary certification with tribal reciprocity in Arizona. Despite many commonalities, foundational differences between CHRs and *promotoras de salud* required stakeholders to embrace the *promotora* experience rooted in the community, as well as that of CHRs who are more directly tied to clinical services. The Arizona experience underscores the benefits of a flexible approach to issues such as defining scope of practice and approving training programs that acknowledges differences in context and leverages existing strengths in organizations and the population served.

## Data Availability

Not applicable

## Abbreviations

CHW: Community health worker
CHR: Community health representative
AzCHOW: Arizona Community Health Worker Association
ADHS: Arizona Department of Health Services
AACIHC: Arizona Advisory Council on Indian Health Care (AACIHC)

## References

[1] Adler NE, Cutler DM, Jonathan J (2016). Addressing social determinants of health and health disparities. Vital Directions for Health and Health Care Initiative: National Academy of Medicine Perspectives.

[2] Frieden TR (2010). A framework for public health action: the health impact pyramid. AJPH..

[3] Marmot M, Friel S, Bell R, Houweling TA, Taylor S (2008). Health. Closing the gap in a generation: Health equity through action on the social determinants of health. Lancet..

[4] Pittman M, Sunderland A, Broderick A, Barnett K (2015). Bringing community health workers into the mainstream of U.S. health care. Discussion Paper.

[5] Kim K, Choi JS, Choi E (2016). Effects of community-based health worker interventions to improve chronic disease management and care among vulnerable populations: a systematic review. AJPH..

[6] Gibbons M, Tyus N (2007). Systematic review of U.S.-based randomized controlled trials using community health workers. Prog Community Health Partner.

[7] Rosenthal EL, Brownstein JN, Rush CH, Hirsch GR, Willaert AM, Scott J (2010). Community health workers: part of the solution. Health Affairs..

[8] Balcazar H, Rosenthal EL, Brownstein JN, Rush CH, Matos S, Hernandez L (2011). Community health workers can be a public health force for change in the United States: three actions for a new paradigm. AJPH..

[9] Sabo S, Flores M, Wennerstrom A (2017). Community health workers promote civic engagement and organizational capacity to impact policy. J Community Health..

[10] Singh P, Chokshi DA (2013). Community health workers—a local solution to a global problem. New Eng J Med..

[11] Ballard M, Schwarz R (2019). Employing practitioner expertise in optimizing community healthcare systems. Healthcare.

[12] Barbero C, Gilchrist S, Chriqui JF (2016). Do state community health worker laws align with best available evidence?. J Community Health..

[13] Centers for Disease Control and Prevention (2014). Policy evidence assessment report. Community health worker policy components.

[14] Chaidez V, Palmer-Wackerly AL, Trout KE (2018). Community health worker employer survey: Perspectives on CHW workforce development in the Midwest. J Community Health..

[15] Association of State and Territorial Health Officials (2018). Variation in state approaches to community health worker certification.

[16] Watts KA (2011). Regulatory moratoria. Duke lJ..

[17] Adakai M, Sandoval-Rosario M, Xu F (2018). Health disparities among American Indians/Alaska Natives - Arizona, 2017. MMWR..

[18] Brown A, Hugo Lopez M (2013). Ranking Latino populations in the nation’s metropolitan areas. Pew Research Center.

[19] Philbin MM, Flake M, Hatzenbuehler ML, Hirsch JS (2018). State-level immigration and immigrant-focused policies as drivers of Latino health disparities in the United States. Soc Sci Med..

[20] Ingram M, Sabo S, Rothers J, Wennerstrom A, de Zapien JG (2008). Community health Workers and community advocacy: addressing health disparities. J Community Health..

[21] Sabo S, Allen CG, Sutkowi K, Wennerstrom A (2017). Community health workers in the United States: challenges in identifying, surveying, and supporting the workforce. AJPH.

[22] Uhrich RB (1969). Tribal community health representatives of the Indian Health Service. Public Health Reports..

[23] Satterfield D, Burd C, Valdez L, Hosey G, Shield JE (2002). The “in-between people”: participation of community health representatives in diabetes prevention and care in American Indian and Alaska Native communities. Health Prom Prac..

[24] Brodt W (1975). Implications for training curriculums from a task inventory survey of Indian community health representatives. Public Health Reports..

[25] Minkler M, Vásquez VB, Tajik M, Petersen D (2008). Promoting environmental justice through community-based participatory research: the role of community and partnership capacity. Health Educ Behav..

[26] Hites LS, Granillo BS, Garrison ER (2012). Emergency preparedness training of tribal community health representatives. J Immigrant Minority Health..

[27] King C, Goldman A, Gampa V (2017). Strengthening the role of community health representatives in the Navajo Nation. BMC Public Health..

[28] Meister J (1989). Guernsey de Zapien J. Un comienzo sano: A model prenatal education project. Health Educ Res..

[29] Warrick L, Wood A, Meister J (1992). al. e. Evaluation of a peer health worker prenatal outreach and education program for Hispanic farmworker families. J Community Health..

[30] Meister J, Giuliano A, Saltzman S, Abrahamsen M, Hunter J, de la Ossa E (1999). Community health workers at the US-Mexico border—effectiveness of a cancer prevention/education intervention. Women & Cancer..

[31] Hunter J, Guernsey de Zapien J, Papenfuss M, Fernandez M, Meister J, Giuliano A (2004). The impact of a Promotora on increasing routine chronic disease prevention among women 40 years of age and older at the US-Mexico border. Health Educ Behav..

[32] Ingram M, Piper R, Kunz S, Navarro C, Sander A, Gastelum S (2012). Salud Si: A case study for the use of participatory evaluation in creating effective and sustainable community-based health promotion. J Fam Community Health..

[33] Ingram M, Torres E, Redondo F, Bradford G, Wang C, O'Toole ML (2007). The impact of promotoras on social support and glycemic control among members of a farmworker community on the US-Mexico border. Diabetes Educ..

[34] Ingram M, Schachter KA, Sabo SJ (2014). A community health worker intervention to address the social determinants of health through policy change. J Prim Prev..

[35] Kunz S, Ingram M, Piper R (2017). Rural collaborative model for diabetes prevention and management: a case study. Health Prom Prac..

[36] Ingram M, Gallegos G, Elenes J (2005). Diabetes is a community issue: the critical elements of a successful outreach and education model on the U.S.-Mexico border. Prev Chron Dis.

[37] Ingram M, Doubleday K, Bell ML (2017). Community health worker impact on chronic disease outcomes within primary care examined using electronic health records. AJPH..

[38] Lohr AM, Ingram M, Nuñez AV, Reinschmidt KM, Carvajal SC (2018). Community–clinical linkages with community health workers in the United States: a scoping review. Health Prom Prac..

[39] American Public Health Association (2009). Community Health Worker Section.

[40] Mason T, Wilkinson GW, Nannini A, Martin CM, Fox DJ, Hirsch GJ (2011). Winning policy change to promote community health workers: lessons from Massachusetts in the health reform era. AJPH..

[41] Malcarney M-B, Pittman P, Quigley L, Seiler N, Horton KB (2015). Community health workers: Health system integration, financing opportunities, and the evolving role of the community health worker in a post-health reform landsacape.

[42] Wilkinson GW, Mason T, Hirsch G (2016). Community health worker integration in health care, public. Health Policy.

[43] Islam N, Shapiro E, Wyatt L (2017). Evaluating community health workers’ attributes, roles, and pathways of action in immigrant communities. Prev Med..

[44] Findley S, Matos S. Bridging the gap: How community health workers promote the health of immigrants. New York: Oxford University Press; 2015.

[45] Sathasivam D, Lupi J, London K. Report to the legislature on community health worker certification: Commonwealth Medicine Publications; 2018. https://escholarship.umassmed.edu/commed_pubs/229. Accessed 2 April 2020.

[46] Warne D, Frizzell LB (2014). American Indian health policy: Historical trends and contemporary issues. AJPH.

[47] Arizona Community Health Worker Association (2019). CHW training program approval guidelines for CHW voluntary certification through the Arizona Community Health Worker Association.

